# Significance of Omega-3 Fatty Acids in the Prophylaxis and Treatment after Spinal Cord Injury in Rodent Models

**DOI:** 10.1155/2020/3164260

**Published:** 2020-07-29

**Authors:** Piotr Wojdasiewicz, Łukasz A. Poniatowski, Paweł Turczyn, Justyna Frasuńska, Agnieszka Paradowska-Gorycka, Beata Tarnacka

**Affiliations:** ^1^Department of Rehabilitation, Eleonora Reicher National Institute of Geriatrics, Rheumatology and Rehabilitation, Spartańska 1, 02-637 Warsaw, Poland; ^2^Department of General and Experimental Pathology, Centre for Preclinical Research and Technology (CePT), Medical University of Warsaw, Pawińskiego 3C, 02-106 Warsaw, Poland; ^3^Department of Experimental and Clinical Pharmacology, Centre for Preclinical Research and Technology (CePT), Medical University of Warsaw, Banacha 1B, 02-097 Warsaw, Poland; ^4^Department of Neurosurgery, Maria Skłodowska-Curie Memorial Cancer Center and Institute of Oncology, W.K. Roentgena 5, 02-781 Warsaw, Poland; ^5^Department of Rehabilitation, Medical University of Warsaw, Faculty of Medicine, Spartańska 1, 02-637 Warsaw, Poland; ^6^Department of Biochemistry and Molecular Biology, Eleonora Reicher National Institute of Geriatrics, Rheumatology and Rehabilitation, Spartańska 1, 02-637 Warsaw, Poland

## Abstract

Polyunsaturated fatty acids (*ω*-3 acids, PUFAs) are essential components of cell membranes in all mammals. A multifactorial beneficial influence of *ω*-3 fatty acids on the health of humans and other mammals has been observed for many years. Therefore, *ω*-3 fatty acids and their function in the prophylaxis and treatment of various pathologies have been subjected to numerous studies. Regarding the documented therapeutic influence of *ω*-3 fatty acids on the nervous and immune systems, the aim of this paper is to present the current state of knowledge and the critical assessment of the role of *ω*-3 fatty acids in the prophylaxis and treatment of spinal cord injury (SCI) in rodent models. The prophylactic properties (pre-SCI) include the stabilization of neuron cell membranes, the reduction of the expression of inflammatory cytokines (IL-1*β*, TNF-*α*, IL-6, and KC/GRO/CINC), the improvement of local blood flow, reduced eicosanoid production, activation of protective intracellular transcription pathways (dependent on RXR, PPAR-*α*, Akt, and CREB), and increased concentration of lipids, glycogen, and oligosaccharides by neurons. On the other hand, the therapeutic properties (post-SCI) include the increased production of endogenous antioxidants such as carnosine and homocarnosine, the maintenance of elevated GSH concentrations at the site of injury, reduced concentrations of oxidative stress marker (MDA), autophagy improvement (via increasing the expression of LC3-II), and p38 MAPK expression reduction in the superficial dorsal horns (limiting the sensation of neuropathic pain). Paradoxically, despite the well-documented protective activity of *ω*-3 acids in rodents with SCI, the research does not offer an answer to the principal question of the optimal dose and treatment duration. Therefore, it is worth emphasizing the role of multicenter rodent studies with the implementation of standards which initially may even be based on arbitrary criteria. Additionally, basing on available research data, the authors of this paper make a careful attempt at referring some of the conclusions to the human population.

## 1. Introduction

Polyunsaturated fatty acids (*ω*-3 acids, PUFAs) are essential components of cell membranes in all mammals [1]. They facilitate normal functioning of the body, but many mammals are unable to synthesize them which necessitates their dietary supply. The group of *ω*-3 includes alpha-linolenic acid (ALA), eicosapentaenoic acid (EPA), and docosahexaenoic acid (DHA) [1, 2]. A multifactorial beneficial influence of *ω*-3 fatty acids on the health of humans and other mammals has been observed for many years. Therefore, *ω*-3 fatty acids and their function in the prophylaxis and treatment of various pathologies have been subjected to numerous studies. The research includes mainly cardiovascular, immune, and nervous system diseases [3–5]. The treatment of patients after spinal cord injury (SCI) constitutes a particular challenge for contemporary medicine, as those injuries primarily contribute to disorders of the nerve tissue and the immune system during inflammation.

Complexity of the natural course of SCI-related pathophysiological events occurs as a result of two subsequent phases such as primary and secondary (delayed) injuries where all parts and compartments of the spinal cord are vulnerable and could be affected [6]. Occurring primary injury is associated with application of external physical force whose character determines the severity of the initial injury [7]. Therefore, primary injury in the case of SCI could in this case result from an external mechanical force (direct/indirect) as well as rapid acceleration/deceleration, ballistic penetration, and less blast exposure arising from shock wave [7]. The macrostructural changes after primary injury in the spinal cord cover damage, laceration, and swelling of the neural tissue as well as various types of structural damage of meninges, ligaments, and bone structures [8]. These events are also connected to the observed changes associated with alternations in circulation of the cerebrospinal fluid (CSF) including an increase of intraspinal pressure (ISP) resulting in subsequent reduction of spinal cord perfusion pressure (SCPP) [9]. The main effects of primary injury cover immediate death of various cell populations associated with rapid reflux of neurotransmitters, dysregulation of transmembrane permeability, and dysfunction of neurovascular units forming blood-spinal cord barrier [10]. Secondary damage includes nonlinear phenomena, including several self-propagating immunology, neurometabolic, and neurochemical events resulting in progressive neurodegeneration [11]. The main mechanisms involved in secondary injury cover local and systemic immunoactivation that involves a multitude of inflammatory mediators and cells, cell death via necrosis or apoptosis, glutamate excitotoxicity, induction of oxidative stress and free-radical generation, altered energy metabolism due to mitochondria dysfunction, and impairment of adenosine-5′-triphosphate (ATP) production and calcium- (Ca^2+^-) mediated neurotoxicity associated with abnormal cell membrane permeability [12]. Both, primary and secondary injury-associated events in all neurostructural levels finally lead to progressive neuronal loss, demyelination, lesion expansion, and glial scar formation which results in neurodegeneration of affected parts of the spinal cord manifesting themselves as neurological deficits due to interruption of axonal connections [13, 14]. SCI should be treated not only as local damage but also as systemic pathology, which is manifested in an immune response involving changes in the transcriptome set, proteome set, immune cell recruitment, and the production of inflammatory mediators [15]. One of the main events in SCI is the activation and proliferation of microglial cells (Iba-1^+^) and astrocytes (GFAP^+^) which together with immune cells participate in the creation of a tightly regulated inflammatory microenvironment [16]. In this case, activated microglia cells and astrocytes serve as one of the main sources of cytokines, proteinases, extracellular matrix molecules (ECMs), and growth factors at the epicenter of lesions [17]. Of all secreted cytokines, both in experimental and clinical conditions, tumor necrosis factor alpha (TNF-*α*) together with interleukin 1 beta (IL-1*β*) and interleukin 6 (IL-6) dominate in the pathogenesis of SCI and other neurological diseases [18].

It is estimated that approximately 6 million patients are affected by SCI worldwide. Those patients suffer from impaired mobility manifesting as paraplegia or tetraplegia [19]. SCI is associated with the occurrence of numerous complications with the most common ones being the infections of the urinary or respiratory system, development of pressure ulcers, cardiovascular disorders, sleep disturbance, depression, muscle atrophy, and osteoporosis [20]. Circumstances in which SCIs occur in people are commonly heterogenous, sudden, and difficult to predict (e.g., traffic accidents and falls from a height) which makes it hard to formulate reliable conclusions from the implemented treatment. It is much easier to introduce reliable conditions of research on SCI on the animal model during controlled laboratory trials. Rodents, such as mice and rats, belong to the group of animals whose physiology of life processes presents numerous similarities to that of humans. Therefore, this kind of research is currently the most preferred.

Regarding the documented therapeutic influence of *ω*-3 fatty acids on the nervous and immune systems [21, 22], the aim of this paper is to present the current state of knowledge and the critical assessment of the role of *ω*-3 fatty acids in the prophylaxis and treatment of SCI in rodent models. Basing on available research data in this area, the authors of this paper make a careful attempt at referring some of the conclusions to the human population.

## 2. The Structure of *ω*-3 Fatty Acids

**Figure 1:**
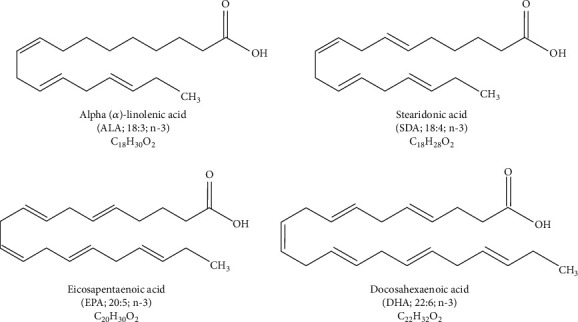
Structure and chemical formulas of *ω*-3 acids.

Fatty acids are characterized by the presence of the carbon chain with a methyl group (-CH_3_) at one end and a carboxyl group (–COOH) at the other [23]. Unsaturated fatty acids are characterized by the carbon chain which includes at least one double bond. Compounds including one double bond are called monounsaturated fatty acids (MUFAs), while those with two or more double bonds, PUFAs [24]. Another criterion of fatty acid classification is the number of carbon atoms in the carbon chain. Short-chain fatty acids contain up to 13 carbon atoms; long-chain ones, between 14 and 19; and very long-chain fatty acids, over 20 carbon atoms. Notably, fatty acids which occur in humans are mainly characterized by the even number of carbon atoms in the carbon chain. *ω*-3 fatty acids are a group of chemical compounds with the common feature—the presence of the last double bond in the carbon chain three carbon atoms away from the -CH_3_ group. They include polyunsaturated fatty acids such as ALA (the carbon chain consists of 18 carbon atoms), EPA (20 carbon atoms in the carbon chain), and DHA (22 carbon atoms in the carbon chain) [23, 24]. Their structure and more, including the location of the double bonds, are presented in Figure 1.

## 3. The Biological Function of *ω*-3 Acids

*ω*-3 acids are mainly present in seafood (lean and oily fish) and in plants—numerous nuts, seeds of oily plants, and green leaves. Fatty acid intake in the Western diet is still dominated by *ω*-6 acids [25]. However, the tendency towards the consumption of *ω*-3 acids is on the rise, because of better health education and the mass production and consumption of dietary supplements based on ALA, EPA, and DHA [25].

The function of *ω*-3 acids has been widely studied and described in professional literature available worldwide. Study results explicitly indicate that *ω*-3 acids present the anti-inflammatory activity, as they reduce the production of inflammatory factors and limit undesirable effects of immune cell activation. It is worth emphasizing that such an activity is of comprehensive and multiplane nature. At the cellular level, the activity of nuclear factor kappa B (NF*κ*B) is reduced [24, 26]. It is one of the most important nuclear transcription factors responsible for the increased expression of genes stimulating inflammatory processes, including those contributing to the synthesis of inflammatory cytokines, chemotactic and adhesion molecules. Several mechanisms may cause the inhibition of NF*κ*B activity. One is associated with the increased expression of peroxisome proliferator-activated receptor gamma (PPAR-*γ*) and genes responsible for the synthesis of agonists of this receptor via the consumption of increased doses of *ω*-3 acids [27]. The activation of PPAR-*γ* and associated signaling pathways inhibits NF*κ*B translocation to the cell nucleus and gene expression in the cell nucleus presenting anti-inflammatory properties [27]. Another mechanism is connected with the ability of fatty acids to activate G-protein-coupled cell membrane receptors. G-protein-coupled receptor 120 (GPR120) is such a receptor, which, thanks to DHA, and probably EPA, demonstrates suppressive properties on the activation of intracellular phosphorylation pathways leading to NF*κ*B activation [28].

The predominant anti-inflammatory activity of *ω*-3 acids consists in reducing the production of eicosanoids (ECS) dependent on arachidonic acid (20:4n-6) such as prostaglandin E2 (PGE_2_) and leukotriene B4 (LTB_4_) and increasing the production of prostaglandin E3 (PGE_3_) and leukotriene B5 (LTB_5_) which present a considerably lower inflammatory activity and are dependent on ALA and EPA [24, 29]. ECS are a group of lipid inflammatory mediators including prostanoids (prostacyclins, thromboxanes, and prostaglandins), leukotrienes, and lipoxins [30]. They are produced as a result of the activity of specific enzymes, cyclooxygenase (COX), lipoxygenase (LOX), and P450 enzymes, with substrates such as arachidonic acid, linoleic acid, or ALA. All human and animal studies confirmed that using high doses of *ω*-3 acids (apart from ALA, EPA, and DHA) diminished the local and systemic inflammatory response via reducing the levels of “strong” ECS in the blood serum (PGE_2_ and LTB_4_ mentioned before). It is linked to the change in the composition of the cell membrane which is due to the reduction in the percentage of *ω*-6 acids included and the increased percentage of *ω*-3 acids [29, 30].

The increased percentage of *ω*-3 acids in the cell membrane may also play a role in inhibiting the development of various neoplastic cells. Some studies showed that *ω*-3 acids inhibited cell divisions in tumors over the synthesis phase (S phase) and prevented the initiation of the second growth phase (G2 phase). Prolonging cell cycle arrest status leads to the apoptosis of neoplastic cells. A potential related mechanism involves the activation of sphingomyelinase (SMYase) on the cell membrane by *ω*-3 acids which increases the synthesis of ceramide [31]. Ceramide, via further activation of phosphatase 1 (PP1) and 2A (PP2A) proteins and p21 protein, inhibits the phosphorylation of the retinoblastoma protein (pRb) in a two-way manner [32], which prevents further cell divisions within the tumor. Interestingly, some authors noted that the above signaling pathways may only lead to the apoptosis of neoplastic cells. As regards healthy cells, e.g., of the retina, heart, or neurons, *ω*-3 acids present protective properties which inhibit programmed cell death [33, 34]. The duality of this activity has not been explained so far. Most probably, it uses the same signaling pathways, but the issue requires further research.

The supplementation with *ω*-3 acids also results in the reduced chemotaxis of neutrophils and monocytes, i.e., the key process responsible for the development of inflammation [35]. The tendency towards reduced chemotaxis occurred for such chemoattractants as ECS (LTB_4_), bacterial antigens, and blood serum proteins. Another key element of the spread of an inflammation apart from the migration of activated inflammatory cells is their ability to pass through the lumen of the blood vessel to the site of inflammation. Regular intake of fish oils including EPA and DHA diminished the expression of intercellular adhesion molecule 1 (ICAM-1) in the membranes of immune cells and vascular cell adhesion molecule 1 (VCAM-1) on the surface of vascular endothelium cells [36]. Such a tendency was observed particularly in monocytes. The molecules are responsible for binding leukocytes to the vascular wall enabling the initiation of their diapedesis to the extravascular space. T cells constitute another cell line influenced by EPA and DHA. *ω*-3 fatty acids decrease their proliferation and reactivity in cell cultures. Most probably, the phenomenon is dose-dependent [21].

EPA and DHA also belong to the substrates of resolvins (EPA, DHA) and protectins (DHA), which are produced via COX and LOX pathways [37–39]. The activity of resolvins and protectins is highly anti-inflammatory. Cell culture and rodent studies demonstrated a multidirectional character of inflammation reduction. Resolvin E1 (RvE1), presenting affinity for RvE1 receptor and leukotriene B1 receptor (BLT_1_) for which it competes with LTB_4_, blocks leukocyte chemotaxis to the site of inflammation and binding to the vascular endothelium cells. Furthermore, binding to N-formyl peptide receptor 2 (FPR2/ALX) and resolvin D1 (RvD1) receptor, it blocks the biological activity of lipoxin A_4_ and annexin A1 and reduces the production of IL-1*β* [38]. Protectin D1 presents an inhibitory effect on the cellular synthesis of IL-1*β* and TNF-*α* which are responsible for increased proinflammatory inducible nitric oxide synthase (iNOS) expression. Apart from limiting the production of IL-1*β* and TNF-*α* by the cells of the vascular endothelium and endoxin-stimulated macrophages and monocytes, it was noted that the supplementation with fish oils at high doses also reduced the synthesis of interleukin 2 (IL-2) and IL-6 [40]. However, the effect was not observable at doses lower than 2 g EPA+DHA day^−1^ [40].

## 4. The Biological Effect of *ω*-3 Acids in Spinal Cord Injury in Rodent Models

Rodents are the most common animal model which is used in research on the influence of *ω*-3 acids on the injured spinal cord. The majority of studies involve iatrogenic spinal cord injury and the observation of changes in the functional and histologic parameters following the supplementation with *ω*-3 acids compared to control groups. Most studies conducted in this way, sometimes enhanced with further modifications, reveal a positive role of *ω*-3 acids on the improvement of the health status in animals with SCI [41–43].

### 4.1. Histological and Anti-inflammatory Improvement after SCI

A study by Lim et al. [41] showed that mice with the *Caenorhabditis elegans* fat-1 gene (fat-1 mice), which is responsible for the increased endogenous production of *ω*-3 acids, were characterized by a more rapid recovery after SCI compared to mice which received a diet low in *ω*-3 acids, standard laboratory diet, or diet with an increased percentage of *ω*-6 acids, respectively. Primarily, fat-1 mice presented a faster recovery of motor functions after SCI. The histological examination of tissues collected from the injury site in this group showed an increased number of neurons and oligodendrocytes with a simultaneous decrease in the number of macrophages and concentrations of inflammatory cytokines. Emon et al. [44] studied the histologic preparations of the nerve tissue collected on day 35 after SCI from rats fed with *ω*-3 acids. They reported reduced spinal cord edema, white matter cavitation, demyelination, and vessel ingrowth. Similar observations were made for mice which received *ω*-3 acids prior to planned SCI, which indicated their prophylactic role in terms of developing an inflammation following neurotrauma. Apart from the above-mentioned *in vivo* research, *in vitro* studies also confirmed the protective influence of *ω*-3 acids on nerve cells and the activation of regenerative processes. A markedly lower concentration of IL-6 and KC/GRO/CINC was noted in fat-1 mice compared to control groups. The authors explained it by the positive effect of *ω*-3 acids on the stabilization of cell membranes, improvement of local blood flow, reduced production of ECS, inhibition of glutamatergic pathways, and the activation of protective intracellular transcription pathways. It is considered to be mainly due to DHA influence which blocks voltage-sensitive Na^+^ and Ca^2+^ channels and activates two-domain background K^+^ channels (e.g., TREK-1, TREK-2, and TRAAK channels). It results in inhibiting the phenomenon of the depolarization-induced increased activation of glutamate receptors [43]. Similar results concerning the anti-inflammatory activity of *ω*-3 acids were obtained by Bi et al. [45], who noted a statistically significant (*p* < 0.05) decrease in the concentrations of proinflammatory IL-6, interleukin 1 receptor antagonist (IL-1ra), C-reactive protein, and TNF-*α* in rats with SCI which were fed with food containing *ω*-3 acids. The concentration of anti-inflammatory interleukin 10 (IL-10) was directly proportional to the *ω*-3/*ω*-6 acid ratio [43].

### 4.2. Antioxidant Activity

Some authors indicated a significant influence of *ω*-3 acids on antioxidant activity during cellular processes following SCI [43, 46]. *ω*-3 acids increased the production of endogenous antioxidants such as carnosine and homocarnosine, which resulted in the diminished synthesis of nitrogen oxide, concentration of immune cells at the site of injury, neuron apoptosis, and the production of inflammatory cytokines. Moreover, the administration of *ω*-3 acids to rats with SCI significantly increased the concentration of antioxidant catalase (CAT) and superoxide dismutase (SOD) reducing the concentration of malondialdehyde (MDA), a typical marker of oxidative stress. *ω*-3 acids diminished the expression of glutamine synthetase which is responsible for reducing the activity of glutamate-induced excitotoxicity in the injured tissue. It was noted that diet rich in *ω*-3 acids maintained the elevated concentrations of glutathione (GSH) at the site of injury. It protected biologically active proteins from irreversible oxidization. Therefore, neutrophil infiltration was limited, and the apoptosis of nerve cells was inhibited at the site of injury, which translated into the improvement of the motor function in rodents with SCI [43, 46].

Another potential benefit associated with using *ω*-3 acids in the diet of rodents with SCI is the increased presence of hemoproteins, such as cytoglobin or neuroglobin, in the nerve tissue [46]. Those hemoproteins improved the intracellular transport of oxygen, simultaneously binding free radicals and nitrogen oxide. The authors pointed out that the precise mechanism of the influence of *ω*-3 acids on the metabolism of hemoproteins had not been elucidated and required further analyses.

### 4.3. Energy Processes Supporting

Interestingly, *ω*-3 acids were found to increase the concentration of lipids, glycogen, and oligosaccharides in the nerve tissue cells [46]. The fact that *ω*-3 acids influence the promotion of glycogenesis and inhibit glycogen disintegration contributes to the increase of energy supplies necessary for healing processes. In view of undisturbed access to energy substrates, neurons are more durable and resistant to inflammatory processes following SCI (apoptosis inhibition). They also demonstrate a tendency towards the increased synthesis of antioxidants and molecules responsible for cell divisions (e.g., amino acids, lipids, and nucleotides). Additional support of post-SCI energy processes which is offered by *ω*-3 acids is connected with the elevated expression of glucose transporter 1 (GLUT-1) and increased synthesis of phosphates in the injured tissue. According to some authors, it has a secondary effect on the increased synthesis of synaptic proteins and the activation of inactive neurons. The resultant increase in nerve tissue plasticity in animal models after SCI was noted both after oral administration and following DHA injections at the site of injury [46]. Notably, it is highly probable that DHA exerts an effect on the activation of transcription pathways associated with the activation of the retinoid X receptor (RXR). Apparently, DHA is a ligand for RXR, which is, in turn, responsible for promoting numerous anabolic processes in tissues, such as cell division, growth, and differentiation of various cell lines. The stimulation of neuroplasticity by *ω*-3 acids may be due to the promotion of an RXR-dependent pathway [43].

### 4.4. Antiapoptotic Properties

*ω*-3 acids also influence epigenetic mechanisms via the increased acetylation of lysine, histone H3, and antiapoptotic B-cell lymphoma 2 (Bcl-2) marker in human neuronal M17 cells. Research conducted on rodent models corroborated this tendency [46]. The increased expression of biological factors inhibiting apoptosis was accompanied by the reduced mRNA expression for proapoptotic proteins, such as Bacillus circulans xylanase (bcx), p53, caspase-3, and pronerve growth factor (pro-NGF) mRNA. Therefore, *ω*-3 acids promoted the expression of neuroprotective genes, which resulted in increasing the resistance of glial cells and neurons to glutamate toxicity, apoptosis, and calcium overload and reduced the motor dysfunction in rats which received *ω*-3 acids before and after SCI.

### 4.5. Antinociceptive Properties

It was also demonstrated that diet richer in *ω*-3 acids reduced thermal hypersensitivity in SCI. An experiment conducted by Figueroa et al. [47] confirmed that the implementation of an 8-week prophylactic diet enriched with *ω*-3 acids prior to and continued for 8 weeks after induced SCI alleviated chronic neuropathic pain in rats. As a result of conversion, the increased concentration of *ω*-3 acids in the cell membranes of neurocytes and glial cells caused an increased synthesis and accumulation of N-acylethanolamine (NAE) precursors such as docosahexaenoyl ethanolamine (DHEA), docosapentaenoyl ethanolamine (DPEA), and eicosapentaenoyl ethanolamine (EPEA). N-Acylethanolamines are a part of the endocannabinoid system which is responsible for numerous life processes, e.g., energy metabolism, immune processes, and, as regards the present study, the inhibition of pain sensation and neuroprotection [48–51]. DHEA, DPEA, and EPEA belong to the class of nuclear factor agonists which inhibit the synthesis of proinflammatory molecules, such as histamine, TNF-*α*, cyclooxygenase 2 (COX-2), and iNOS, via binding with proliferator activated receptor alpha (PPAR-*α*) in the cell nucleus. Furthermore, DHEA and EPEA present affinity for cannabinoid receptors and DPEA—for cannabinoid-like G-coupled receptors GPR55 and GPR119. Therefore, it is claimed that DHEA, DPEA, and EPEA present antinociceptive, anticonvulsant, anti-inflammatory, and neuroprotective properties in SCI. It was also observed that the expression of p38 mitogen-activated protein kinase (p38 MAPK) in the superficial dorsal horns was significantly reduced in the nerve tissue in rats which received diet enriched with *ω*-3 acids. p38 MAPK is responsible for processes associated with the sensation of neuropathic pain [52, 53]. It was also noted that diet rich in *ω*-3 acids decreased the rate of the regenerative processes of nociceptive fibers, which was indirectly assessed by the evaluation of the expression of calcitonin gene-related peptide (CGRP) at the site located 3-5 mm below SCI. Therefore, reducing the expression of p38 MAPK and CGRP in the nerve tissue made the rats tend to recover more rapidly after SCI thanks to the alleviation of neuropathic pain.

### 4.6. Nerve Regeneration

Prophylactic diet enriched with *ω*-3 acids also significantly changed the concentrations of proteins participating in the promotion of neuroplasticity and the regeneration of nerve cells from tissues collected from rats with SCI, i.e., protein kinase B (Akt) and cAMP-response element binding (CREB) protein [54, 55]. Both proteins are a part of intracellular signaling pathways which activate the synthesis of mRNA specific for cell regeneration in the cell nucleus. CREB functions include the regulation of crucial cell stages (proliferation, differentiation, and survival) [56]. Akt is a critical mediator of growth factor-induced neuronal survival which participates in inhibiting apoptosis [57].

An improvement in motor function recovery in rats with SCI at T10 segment induced with high *ω*-3 acid diet was confirmed in a study by Nie et al. [58]. Compared to control groups, rats which received 29.75 g/kg of dietary EPA and 17.19 g/kg of dietary DHA presented an increased expression of microtubule-associated protein 1A/1B-light chain 3 type II (LC3-II) after a week and two weeks following the operation. LC3-II is recruited to autophagosomal membranes. Therefore, it is thought that labeling the expression of LC3-II refers to the intensification of tissue autophagy. In case of SCI, it is considered that effective autophagy at the site of injury is beneficial—effective removal of damaged tissue facilitates faster initiation of regenerative processes. *In vitro* studies confirmed that an increased expression of LC3-II is due to the inhibition of the mammalian target of rapamycin complex 1 (mTORC1) signaling pathway. Mammalian target of rapamycin (mTOR) is a serine/threonine kinase which participates in the regulation of metabolism, proliferation, growth, and autophagy of cells. Studies showed that inhibiting the activity of mTORC1 increased autophagy and accelerated regenerative processes in tissues [59, 60]. The activity of *ω*-3 acids decreased the expression of mTORC1 in neurons which made them much more susceptible to repair processes following an injury. Reduced mTORC1 expression is also influenced by lowered concentrations of protein S6 (p-S6) and protein S6 kinase (p-S6K) in tissues which is also triggered by *ω*-3 acids. Under clinical conditions, it manifested as a more rapid recovery of hindlimb motor function, while the electrophysiologic monitoring of the high *ω*-3 acid diet group demonstrated a statistically lower latency and a higher amplitude of motor evoked potential. It translated into a statistically significant improvement in the function of the urinary bladder and locomotor function score according to the BBB scale (Table 1) [61] and better results of walking on a horizontal ladder or beam [62].

## 5. Summary and Further Perspectives

**Figure 2:**
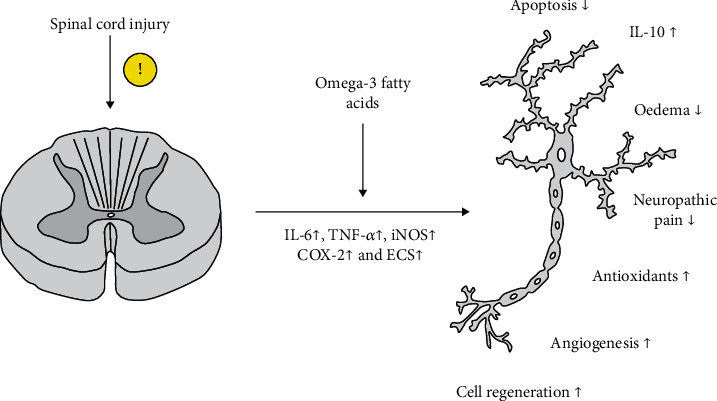
Presentation of the effect of *ω*-3 acids on nervous tissue after spinal cord injury in rodent models. Explanation of abbreviations in the text.

The above-mentioned results of studies conducted on the rodent model indicate that *ω*-3 acids present both prophylactic and therapeutic properties towards the nervous tissue affected by SCI (Figure 2).

The prophylactic properties (pre-SCI status) of *ω*-3 acids which significantly improved the outcomes in rodents following iatrogenic SCI include the stabilization of neuron cell membranes; the reduction of the expression of inflammatory molecules such as IL-6, KC/GRO/CINC, IL-1ra, C-reactive protein, and TNF-*α*; the improvement of local blood flow; reduced ECS production; the inhibition of glutamatergic pathways; the activation of protective intracellular transcription pathways dependent on RXR, PPAR-*α*, Akt, and CREB; and increased concentration of lipids, glycogen, and oligosaccharides by neurons.

The therapeutic properties (post-SCI status) include the above-mentioned factors from the list of prophylactic ones and the increased production of endogenous antioxidants such as carnosine and homocarnosine, reduced concentrations of oxidative stress marker (MDA), and the maintenance of elevated GSH concentrations at the site of injury, which protects proteins from oxidization. Moreover, the therapeutic properties include autophagy improvement (via increasing the expression of LC3-II) and p38 MAPK expression reduction in the superficial dorsal horns (limiting the sensation of neuropathic pain).

A summary of *ω*-3 acid properties after SCI is collected and presented in Table 2.

Prophylactic and therapeutic activity significantly improves urinary bladder functions, locomotor function score according to the BBB scale, and gait results. Notably, the results of therapeutic actions are poorer than those of the use of prophylaxis only or prophylaxis with *ω*-3 acid administration following SCI.

According to the majority of studies, the locomotor function outcomes depend on the dose of *ω*-3 acids added to the diet of rodents. Higher doses translated into a more rapid and effective recovery presented by study animals. The analyses did not comprise the maximum acceptable dose in the rodent diet after which further improvement of post-SCI health status was no longer observed. It is a partially individual issue, but it causes difficulties in the assessment of the final influence of *ω*-3 acids on the improvement of post-SCI neurological status. No data are available to answer the important question of whether the status might deteriorate at some higher doses of *ω*-3 acids.

The research also did not provide response concerning the optimal duration of pre-SCI prophylaxis with *ω*-3 acids. In many cases, it is arbitrarily set between 7 days and even 8 weeks [47, 48]. The improvement of motor functions in rodents resulted from the implementation of *ω*-3 acid-enriched diet as short as 7 days. However, it was not determined what the optimal duration of supplementation may be and, if prolonged, if there would be any statistical difference in the results.

As demonstrated, despite the explicit results of the above-mentioned studies indicating the positive impact of *ω*-3 acids on numerous aspects of recovery in rodents with SCI, standards regarding the dose and duration of such treatment have not been developed yet. It is known that rodent studies constitute an introduction to the assessment of therapy outcomes in humans. Therefore, it is notable how much difficulty researchers face when attempting to determine the effectiveness of high doses of *ω*-3 acids used in the diet of SCI patients. It is justified to expect that diet enriched with *ω*-3 acid may have protective and anabolic properties towards the nerve tissue after an injury. However, the determination of the optimal therapeutic dose may be even more difficult than in rodents. SCI patients are a highly heterogenous group as regards diet, weight, drug history, and the genotype. The deficit of *ω*-3 acids in their diet and the time of diet saturation are hard to assess. The obtained data may be unreliable as regards certain doses. The optimal model of the assessment of the effect of *ω*-3 acids on nerve tissue regeneration and an improvement of locomotor functions in patients with SCI may be based on selecting patients who received prophylactic doses of *ω*-3 acids (at least 2 g EPA+DHA day^−1^) [40]. However, such a habit of supplementation in a randomly completed group is rare, as people usually do not anticipate an injury.

Paradoxically, despite the well-documented protective activity of *ω*-3 acids in rodents with SCI, the research does not offer an answer to the principal question of the optimal dose and treatment duration. Each new analysis provides new information concerning the effect of *ω*-3 acids on cellular pathways, secretion of cytokines, cell hormones, or the interactions with cell receptors. However, the effective implementation of this knowledge remains challenging. Therefore, it is worth emphasizing the role of multicenter rodent studies with the implementation of standards which initially may even be based on arbitrary criteria. It is possible that such studies may facilitate the development of an effective therapy with *ω*-3 acids in humans from a longer perspective. Neglecting the well-documented available research on the therapeutic potential of *ω*-3 acids would be a great waste.

## Figures and Tables

**Table 1:** Basso, Beattie, Bresnahan (BBB) locomotor rating scale. BBB scale is used as a tool to assess locomotor recovery after spinal cord injury in rodent models [48].

Points	Clinical observation
0	No observable movement of the hindlimbs.
1	Slight (limited) movement of one or two joints, usually hip and/or knee.
2	Extensive movement of one joint or extensive movement of one joint and slight movement of the other.
3	Extensive movement of two joints.
4	Slight movement of all three joints of the hindlimbs.
5	Slight movement of two joints and extensive movement of the third joint.
6	Extensive movement of two joints and slight movement of the third joint.
7	Extensive movement of the three joints in the hindlimbs.
8	Sweeping without weight-bearing or plantar support of the paw without weight-bearing.
9	Plantar support of the paw with weight-bearing only in the support stage (i.e., when static) or occasional, frequent, or inconsistent dorsal stepping with weight-bearing and no plantar stepping.
10	Plantar stepping with occasional weight-bearing and no forelimb-hindlimb coordination.
11	Plantar stepping with frequent to consistent weight-bearing and occasional forelimb-hindlimb coordination.
12	Plantar stepping with frequent to consistent weight-bearing and occasional forelimb-hindlimb coordination.
13	Plantar stepping with frequent to consistent weight-bearing and frequent forelimb-hindlimb coordination.
14	Plantar stepping with consistent weight support, consistent forelimb-hindlimb coordination, and predominantly rotated paw position (internally or externally) during locomotion both at the instant of initial contact with the surface as well as before moving the toes at the end of the support stage or frequent plantar stepping, consistent forelimb-hindlimb coordination, and occasional dorsal stepping.
15	Consistent plantar stepping, consistent forelimb-hindlimb coordination, and no movement of the toes or occasional movement during forward movement of limb; predominant paw position is parallel to the body at the time of initial contact.
16	Consistent plantar stepping and forelimb-hindlimb coordination during gait and movement of the toes occurring frequently during forward movement of the limb; the predominant paw position is parallel to the body at the time of initial contact and curved at the instant of movement.
17	Consistent plantar stepping and forelimb-hindlimb coordination during gait and movement of the toes occurring frequently during forward movement of limb; the predominant paw position is parallel to the body at the time of initial contact and at the instant of movement of the toes.
18	Consistent plantar stepping and forelimb-hindlimb coordination during gait and movement of the toes occurring consistently during forward movement of limb; the predominant paw position is parallel to the body at the time of initial contact and curved during movement of the toes.
19	Consistent plantar stepping and forelimb-hindlimb coordination during gait and movement of the toes occurring consistently during forward movement of limb; the predominant paw position is parallel to the body at the instant of contact and at the time of movement of the toes, and the animal presents a downward tail some or all of the time.
20	Consistent plantar stepping and forelimb-hindlimb coordination during gait and movement of the toes occurring consistently during forward movement of limb; the predominant paw position is parallel to the body at the instant of contact and at the time of movement of toes, and the animal presents consistent elevation of the tail and trunk instability.
21	Consistent plantar stepping and coordinated gait, consistent movement of the toes; paw position is predominantly parallel to the body during the whole support stage; consistent trunk stability; consistent tail elevation.

**Table 2:** Summary of the biological effects of *ω*-3 fatty acids on cellular processes and clinical outcome after spinal cord injury in rodent models. Explanation of abbreviations in the text.

Study	Biological effects of *ω*-3 fatty acids in rodent models after SCI
Mortazavi et al. [10]	↓ Concentrations of IL-6, KC/GRO/CINC
	↓ Number of macrophages
	↓ ECS production
	Inhibition of glutamatergic pathways
	Stabilization of cell membranes
	↑ Local blood flow
	↑ Number of neurons
	↑ Number of oligodendrocytes

Borgens and Liu-Snyder [12]	Inhibition of voltage-sensitive Na^+^ and Ca^2+^ channels
	Activation of two-domain background K^+^ channels (TREK-1, TREK-2, and TRAAK channels)
	Inhibition of the depolarization-induced increased activation of glutamate receptors
	The activation of transcription pathways associated with the activation of retinoid X receptor (RXR)
	↑ Neuroplasticity
	↑ Concentrations of IL-10

Kawano et al. [13]	↓ Spinal cord edema
	↓ White matter cavitation
	↓ Demyelination
	↓ Vessel ingrowth

Yuan and He [14]	↓ Concentrations of IL-6, IL-1ra, C-reactive protein, and TNF-*α*

Sun et al. [15]	↑ Expression of cytoglobin and neuroglobin
	↑ Concentration of lipids, glycogen, and oligosaccharides by neurons
	↑ Expression of glucose transporter 1 (GLUT-1)
	↑ Synthesis of phosphates
	↑ Synthesis of synaptic proteins
	↑ Acetylation of lysine, histone H3, and antiapoptotic Bcl-2 marker in human neuronal M17 cells
	↑ Resistance of glial cells and neurons on the activity of glutamate toxicity, apoptosis, and calcium overload
	↓ Expression of mRNA for bcx, p53, caspase-3, and pro-NGF
	Activation of inactive neurons

Beck et al. [16]	↓ Thermal hypersensitivity
	↓ Synthesis of histamine, TNF-*α*, COX-2, and iNOS
	↓ Expression of p38 mitogen-activated protein kinase
	↑ Expression of B/Akt (Akt) and CREB
	↑ Synthesis and accumulation of the precursors of N-acylethanolamines (NAE): DHEA, DPEA, and EPEA

Belchior et al. [27]	↑ Expression of microtubule-associated protein 1A/1B-light chain 3 type II (LC3-II)
	↓ Expression of mammalian target of rapamycin complex 1 (mTORC1) signaling pathway
	↓ Concentrations of p-S6 and p-S6K

## References

[1] Shahidi F., Ambigaipalan P. (2018). Omega-3 polyunsaturated fatty acids and their health benefits. *Annual Review of Food Science and Technology*.

[2] Cholewski M., Tomczykowa M., Tomczyk M. (2018). A comprehensive review of chemistry, sources and bioavailability of omega-3 fatty acids. *Nutrients*.

[3] Abdelhamid A. S., Brown T. J., Brainard J. S. Omega-3 fatty acids for the primary and secondary prevention of cardiovascular disease. *Cochrane Database of Systematic Reviews*.

[4] Avallone R., Vitale G., Bertolotti M. (2019). Omega-3 fatty acids and neurodegenerative diseases: new evidence in clinical trials. *International Journal of Molecular Sciences*.

[5] Li X., Bi X., Wang S., Zhang Z., Li F., Zhao A. Z. (2019). Therapeutic potential of *ω*-3 polyunsaturated fatty acids in human autoimmune diseases. *Frontiers in Immunology*.

[6] Tator C. H., Fehlings M. G. (1991). Review of the secondary injury theory of acute spinal cord trauma with emphasis on vascular mechanisms. *Journal of Neurosurgery*.

[7] Silva N. A., Sousa N., Reis R. L., Salgado A. J. (2014). From basics to clinical: a comprehensive review on spinal cord injury. *Progress in Neurobiology*.

[8] Dumont R. J., Okonkwo D. O., Verma S. (2001). Acute spinal cord injury, part I: pathophysiologic mechanisms. *Clinical Neuropharmacology*.

[9] Varsos G. V., Werndle M. C., Czosnyka Z. H. (2015). Intraspinal pressure and spinal cord perfusion pressure after spinal cord injury: an observational study. *Journal of Neurosurgery. Spine*.

[10] Mortazavi M. M., Verma K., Harmon O. A. (2015). The microanatomy of spinal cord injury: a review. *Clinical Anatomy*.

[11] Oyinbo C. A. (2011). Secondary injury mechanisms in traumatic spinal cord injury: a nugget of this multiply cascade. *Acta Neurobiologiae Experimentalis (Wars)*.

[12] Borgens R. B., Liu-Snyder P. (2012). Understanding secondary injury. *The Quarterly Review of Biology*.

[13] Kawano H., Kimura-Kuroda J., Komuta Y. (2012). Role of the lesion scar in the response to damage and repair of the central nervous system. *Cell and Tissue Research*.

[14] Yuan Y. M., He C. (2013). The glial scar in spinal cord injury and repair. *Neuroscience Bulletin*.

[15] Sun X., Jones Z. B., Chen X. M., Zhou L., So K. F., Ren Y. (2016). Multiple organ dysfunction and systemic inflammation after spinal cord injury: a complex relationship. *Journal of Neuroinflammation*.

[16] Beck K. D., Nguyen H. X., Galvan M. D., Salazar D. L., Woodruff T. M., Anderson A. J. (2010). Quantitative analysis of cellular inflammation after traumatic spinal cord injury: evidence for a multiphasic inflammatory response in the acute to chronic environment. *Brain*.

[17] Loane D. J., Byrnes K. R. (2010). Role of microglia in neurotrauma. *Neurotherapeutics*.

[18] Patterson Z. R., Holahan M. R. (2012). Understanding the neuroinflammatory response following concussion to develop treatment strategies. *Frontiers in Cellular Neuroscience*.

[19] Tan C. O. (2013). Spinal Cord Injury and Osteoporosis: Causes, Mechanisms, and Rehabilitation Strategies. *International Journal of Physical Medicine & Rehabilitation*.

[20] Sezer N., Akkuş S., Uğurlu F. G. (2015). Chronic complications of spinal cord injury. *World Journal of Orthopedics*.

[21] Gutiérrez S., Svahn S. L., Johansson M. E. (2019). Effects of omega-3 fatty acids on immune cells. *International Journal of Molecular Sciences*.

[22] Alnahdi H. S., Sharaf I. A. (2019). Possible prophylactic effect of omega-3 fatty acids on cadmium-induced neurotoxicity in rats' brains. *Environmental Science and Pollution Research International*.

[23] Yashodhara B. M., Umakanth S., Pappachan J. M., Bhat S. K., Kamath R., Choo B. H. (2009). Omega-3 fatty acids: a comprehensive review of their role in health and disease. *Postgraduate Medical Journal*.

[24] Calder P. C. (2017). Omega-3 fatty acids and inflammatory processes: from molecules to man. *Biochemical Society Transactions*.

[25] Ruxton C. (2004). Health benefits of omega-3 fatty acids. *Nursing Standard*.

[26] Daak A. A., Elderdery A. Y., Elbashir L. M. (2015). Omega 3 (n − 3) fatty acids down-regulate nuclear factor-kappa B (NF-*κ*B) gene and blood cell adhesion molecule expression in patients with homozygous sickle cell disease. *Blood Cells, Molecules, and Diseases*.

[27] Belchior T., Paschoal V. A., Magdalon J. (2015). Omega-3 fatty acids protect from diet-induced obesity, glucose intolerance, and adipose tissue inflammation through PPAR*γ*-dependent and PPAR*γ*-independent actions. *Molecular Nutrition & Food Research*.

[28] Oh D. Y., Talukdar S., Bae E. J. (2010). GPR120 is an omega-3 fatty acid receptor mediating potent anti-inflammatory and insulin-sensitizing effects. *Cell*.

[29] Wall R., Ross R. P., Fitzgerald G. F., Stanton C. (2010). Fatty acids from fish: the anti-inflammatory potential of long-chain omega-3 fatty acids. *Nutrition Reviews*.

[30] Khanapure S., Garvey D., Janero D., Gordon Letts L. (2007). Eicosanoids in Inflammation: Biosynthesis, Pharmacology, and Therapeutic Frontiers. *Current Topics in Medicinal Chemistry*.

[31] Wu M., Harvey K. A., Ruzmetov N. (2005). Omega-3 polyunsaturated fatty acids attenuate breast cancer growth through activation of a neutral sphingomyelinase-mediated pathway. *International Journal of Cancer*.

[32] Siddiqui R. A., Shaikh S. R., Sech L. A., Yount H. R., Stillwell W., Zaloga G. P. (2004). Omega 3-fatty acids: health benefits and cellular mechanisms of action. *Mini Reviews in Medicinal Chemistry*.

[33] Jolly C. A., Jiang Y.-H., Chapkin R. S., McMurray D. N. (1997). Dietary (n-3) Polyunsaturated Fatty Acids Suppress Murine Lymphoproliferation, Interleukin-2 Secretion, and the Formation of Diacylglycerol and Ceramide. *The Journal of Nutrition*.

[34] Opreanu M., Lydic T. A., Reid G. E., McSorley K. M., Esselman W. J., Busik J. V. (2010). Inhibition of cytokine signaling in human retinal endothelial cells through downregulation of sphingomyelinases by docosahexaenoic acid. *Investigative Ophthalmology & Visual Science*.

[35] Calder P. C. (2010). Omega-3 fatty acids and inflammatory processes. *Nutrients*.

[36] Goua M., Mulgrew S., Frank J., Rees D., Sneddon A. A., Wahle K. W. J. (2008). Regulation of adhesion molecule expression in human endothelial and smooth muscle cells by omega-3 fatty acids and conjugated linoleic acids: involvement of the transcription factor NF-*κ*B?. *Prostaglandins, Leukotrienes, and Essential Fatty Acids*.

[37] Moro K., Nagahashi M., Ramanathan R., Takabe K., Wakai T. (2016). Resolvins and omega three polyunsaturated fatty acids: clinical implications in inflammatory diseases and cancer. *World Journal of Clinical Cases*.

[38] Krishnamoorthy S., Recchiuti A., Chiang N., Fredman G., Serhan C. N. (2012). Resolvin D1 receptor stereoselectivity and regulation of inflammation and proresolving microRNAs. *The American Journal of Pathology*.

[39] Weylandt K. H., Chiu C.-Y., Gomolka B., Waechter S. F., Wiedenmann B. (2012). Omega-3 fatty acids and their lipid mediators: towards an understanding of resolvin and protectin formation. *Prostaglandins & Other Lipid Mediators*.

[40] Calder P. C. (2013). Omega-3 polyunsaturated fatty acids and inflammatory processes: nutrition or pharmacology?. *British Journal of Clinical Pharmacology*.

[41] Lim S.-N., Gladman S. J., Dyall S. C. (2013). Transgenic mice with high endogenous omega-3 fatty acids are protected from spinal cord injury. *Neurobiology of Disease*.

[42] Satkunendrarajah K., Fehlings M. G. (2013). Do omega-3 polyunsaturated fatty acids ameliorate spinal cord injury?: Commentary on: Lim et al., Improved outcome after spinal cord compression injury in mice treated with docosahexaeonic acid. Exp. Neurol. Jan; 239:13–27. *Experimental Neurology*.

[43] Michael-Titus A. T., Priestley J. V. (2014). Omega-3 fatty acids and traumatic neurological injury: from neuroprotection to neuroplasticity?. *Trends in Neurosciences*.

[44] Emon S. T., Irban A. G., Bozkurt S. U., Akakin D., Konya D., Ozgen S. (2010). Effects of parenteral nutritional support with fish-oil emulsion on spinal cord recovery in rats with traumatic spinal cord injury. *Turkish Neurosurgery*.

[45] Bi J., Chen C., Sun P., Tan H., Feng F., Shen J. (2019). Neuroprotective effect of omega-3 fatty acids on spinal cord injury induced rats. *Brain and Behavior*.

[46] Figueroa J. D., De Leon M. (2014). Neurorestorative targets of dietary long-chain omega-3 fatty acids in neurological injury. *Molecular Neurobiology*.

[47] Figueroa J. D., Cordero K., Serrano-Illan M. (2013). Metabolomics uncovers dietary omega-3 fatty acid-derived metabolites implicated in anti-nociceptive responses after experimental spinal cord injury. *Neuroscience*.

[48] Jackson S. J., Pryce G., Diemel L. T., Cuzner M. L., Baker D. (2005). Cannabinoid-receptor 1 null mice are susceptible to neurofilament damage and caspase 3 activation. *Neuroscience*.

[49] Beltramo M., Bernardini N., Bertorelli R. (2006). CB2 receptor-mediated antihyperalgesia: possible direct involvement of neural mechanisms. *The European journal of neuroscience.*.

[50] Petrosino S., Palazzo E., de Novellis V. (2007). Changes in spinal and supraspinal endocannabinoid levels in neuropathic rats. *Neuropharmacology*.

[51] Hama A., Sagen J. (2011). Activation of spinal and supraspinal cannabinoid-1 receptors leads to antinociception in a rat model of neuropathic spinal cord injury pain. *Brain research.*.

[52] Crown E. D., Gwak Y. S., Ye Z., Johnson K. M., Hulsebosch C. E. (2008). Activation of p38 MAP kinase is involved in central neuropathic pain following spinal cord injury. *Experimental neurology.*.

[53] Gwak Y. S., Unabia G. C., Hulsebosch C. E. (2009). Activation of p-38alpha MAPK contributes to neuronal hyperexcitability in caudal regions remote from spinal cord injury. *Experimental neurology.*.

[54] Figueroa J. D., Cordero K., Baldeosingh K. (2012). Docosahexaenoic acid pretreatment confers protection and functional improvements after acute spinal cord injury in adult rats. *Journal of Neurotrauma*.

[55] Akbar M., Calderon F., Wen Z., Kim H.-Y. Docosahexaenoic acid: a positive modulator of Akt signaling in neuronal survival.

[56] Ortega-Martínez S. (2015). A new perspective on the role of the CREB family of transcription factors in memory consolidation via adult hippocampal neurogenesis. *Frontiers in Molecular Neuroscience*.

[57] Szymonowicz K., Oeck S., Malewicz N. M., Jendrossek V. (2018). New Insights into Protein Kinase B/Akt Signaling: Role of Localized Akt Activation and Compartment-Specific Target Proteins for the Cellular Radiation Response. *Cancers*.

[58] Nie J., Chen J., Yang J. (2018). Inhibition of mammalian target of rapamycin complex 1 signaling by n-3 polyunsaturated fatty acids promotes locomotor recovery after spinal cord injury. *Molecular Medicine Reports*.

[59] Rabanal-Ruiz Y., Otten E. G., Korolchuk V. I. (2017). mTORC1 as the main gateway to autophagy. *Essays in Biochemistry*.

[60] Noda T. (2017). Regulation of autophagy through TORC1 and mTORC1. *Biomolecules*.

[61] Basso D. M., Beattie M. S., Bresnahan J. C. (1995). A sensitive and reliable locomotor rating scale for open field testing in rats. *Journal of Neurotrauma*.

[62] Figueroa J. D., Cordero K., Llán M. S., De Leon M. (2013). Dietary omega-3 polyunsaturated fatty acids improve the neurolipidome and restore the DHA status while promoting functional recovery after experimental spinal cord injury. *Journal of Neurotrauma*.

